# Structural and functional disconnections in non-acute post-stroke patients

**DOI:** 10.3389/fneur.2025.1542292

**Published:** 2025-06-25

**Authors:** Yanan Wu, Chuanshuai Tian, Zhixuan Yu, Zaixing Liu, Han Wu, Jie Ming, Wenjun Hong, Rong Xu

**Affiliations:** ^1^Department of Rehabilitation Medicine, Nanjing Drum Tower Hospital, Affiliated Hospital of Medical School, Nanjing University, Nanjing, China; ^2^Department of Radiology, Nanjing Drum Tower Hospital, Affiliated Hospital of Medical School, Nanjing University, Nanjing, China; ^3^Medical Imaging Center, Affiliated Cancer Hospital of Xinjiang Medical University, Xinjiang, China

**Keywords:** stroke, motor dysfunctions, magnetic resonance imaging, structural connectivity, functional connectivity

## Abstract

**Background:**

Structural alterations and functional reorganizations related to motor dysfunction after stroke remain unclear. This study aims to investigate alterations in structural connectivity (SC) and functional connectivity (FC) in non-acute post-stroke patients, and their associations with motor performance.

**Methods:**

Thirty-six non-acute post-stroke patients and thirty-eight well-matched healthy controls (HCs) were included. SC and FC differences between groups were analyzed using diffusion tensor imaging and resting-state fMRI, respectively. Correlations between SC and FC in regions with significant intergroup differences, along with their correlations with motor performance, were assessed.

**Results:**

Compared with HCs, significant decreases in both SC and FC were observed in stroke patients among the right precentral gyrus, right superior frontal gyrus, right supplementary motor area, right paracentral lobule, right middle cingulate gyrus, right superior marginal gyrus, right middle temporal gyrus, and left inferior temporal gyrus. A negative correlation of SC-FC was found between the right middle cingulate gyrus and right paracentral lobule in stroke group, while a positive correlation was found between the right superior marginal gyrus and right middle temporal gyrus. Moreover, the FC between the right superior marginal gyrus and right middle temporal gyrus showed negative correlations with the Fugl-Meyer assessment of the Upper/Lower Extremity scores.

**Discussion:**

This study identified disconnections in both SC and FC in sensorimotor-related and high-order brain regions, which may enhance understanding of the structure–function interactions underlying motor deficits in post-stroke patients.

## 1 Introduction

Most stroke survivors suffer from varying degrees of hemiplegia (1, 2). About 80% of post-stroke patients will experience upper limb hemiplegia (2, 3), and over 50% will survive lower limb hemiplegia (4, 5). Motor impairments seriously reduce patient independence in activities of daily living and impose a considerable economic burden on families and society (6). While early post-stroke brain functional reorganization does contribute to spontaneous recovery of motor function to some extent (7, 8), the process is usually incomplete and varies significantly among individuals (9). Recent studies suggest that improvements in motor function are still possible during the non-acute stroke phase (10–14). However, the neural mechanism underlying movement dysfunctions in non-acute post-stroke patients remain unclear. Further exploration is needed to identify the brain regions with structural changes and functional reorganization, as well as their relationships with motor dysfunctions.

Recent advances in neuroimaging modalities, particularly multimodal MRI, have facilitated the investigation of structural and functional changes in the brains of post-stroke patients (15, 16). Structural connectivity (SC) refers to the brain’s anatomical organization through fiber tracts, and functional connectivity (FC) represents the statistical dependencies between time series of electro-physiological activity and (de)oxygenated blood levels in distinct brain regions (17). Recent studies utilizing SC and FC have revealed patterns of structural alterations and functional reorganization in stroke patients. For example, Yang et al. found a significant decrease in FC in the right superior frontal gyrus (SFG) of post-stroke patients (18). Paul et al. suggested that motor performances, both basal and complex, was correlated with SC between the bilateral premotor areas and the ipsilesional primary motor cortex, as well as interhemispheric connectivity between the primary motor cortex (M1) (19). Furthermore, Lee et al. observed decreased SC in the contralesional supplementary motor area (SMA), dorsolateral prefrontal cortex, and M1, along with increased FC in these regions in post-stroke patients with motor impairment (20). Our prior studies found structural changes and functional reorganization in the frontoparietal regions and temporal areas (10–12, 21, 22), such as the precentral gyrus (PreCG), SMA, inferior frontal gyrus, superior temporal gyrus, middle temporal gyrus (MTG), middle frontal gyrus and SFG, in non-acute stroke patients with motor dysfunctions. These findings suggest that structural and functional alterations occur not only in sensorimotor-related regions but also in high-order regions among non-acute post-stroke patients with motor disorders (18–20). However, most of previous studies have focused on either SC or FC to identify changes in brain regions after stroke, and combining them to explore brain remodeling patterns in greater depth has not been sufficient.

Combining SC and FC analysis in post-stroke patients with motor impairment enhances the understanding of brain remodeling mechanisms (22, 23). Yu et al. have recently demonstrated that covarying structures and functions related to the ipsilesional M1, dorsal premotor area and primary somatosensory cortex, were positively correlated with the motor performance of the upper extremity in post-stroke patients (24). Zhang et al. observed that the strength of SC-FC coupling in motor- and cognition-related regions, such as the SMA and the posterior cingulate gyrus, was significantly decreased in non-acute post-stroke patients compared with the healthy controls (HCs), and positively correlated with motor function in hemiplegic limbs (25). Similarly, Kalinosk et al. reported a significant decrease in SC-FC coupling in the frontoparietal cortex and cingulate gyrus, which was also positively correlated with motor function (26). These findings highlight the importance of integrating brain structural changes and functional reorganization to elucidate the neural mechanisms of motor dysfunctions in non-acute stroke patients, not only in primary sensorimotor areas, but also in higher cognitive-related regions. However, it is important to note that SC-FC coupling only reflects the correlation between SC and FC with respect to a single brain region or network and does not capture the interactions between different brain regions (27). Analyzing the brain regions with concurrent alterations in both SC and FC provides important insights into understanding the interactions among different brain regions. Despite its potential, research on brain regions with concurrent SC and FC alterations in non-acute post-stroke patients with movement disorders remains limited.

The present study aims to explore the alteration of SC and FC in non-acute post-stroke patients with hemiplegia and its relationship to motor performance using multimodal MRI. Based on findings of structural and functional changes in post-stroke patients (24–26), we hypothesized: (1) SC and FC alterations would occur not only in sensorimotor-related regions but also in high-order regions; (2) these SC and FC alterations were correlated with motor performance in the hemiplegic limb. Understanding these alterations of SC and FC could provide important insights into the pathophysiological mechanisms of non-acute post-stroke and their relationship to motor outcomes.

## 2 Materials and methods

### 2.1 Participants

The study was approved by the Ethics Committee of Nanjing Drum Tower Hospital, Affiliated Hospital of Medical School, Nanjing University, and conducted in accordance with the Declaration of Helsinki. Written informed consent was obtained from all participants or their legal guardians, as appropriate, before participation. All data used in this study were obtained from a research trial registered in the Clinical-Trials.gov database (NCT05648552).

This study recruited 76 eligible participants from Nanjing Drum Tower Hospital and nearby residential areas, comprising 38 non-acute subcortical post-stroke patients with hemiplegia and 38 well-matched HCs. The sample size referred to previous studies investigating structural and functional alterations in post-stroke patients (24, 28). Furthermore, G*Power software^1^ was applied to evaluate the sample size. A *post-hoc* power analysis was calculated for a two-tailed analysis, with an effect size of 0.5, *α* set at 0.05, and *d* set at 0.8, yielding a statistical power (1-*β*) of 0.99.

The inclusion criteria for post-stroke participants were: (1) first-episode subcortical stroke confirmed by CT or MRI; (2) age between 30 and 75 years; (3) right-handedness prior to the stroke; (4) disease duration of stroke ≥ 3 months. The exclusion criteria included: (1) contraindications for MRI; (2) the presence of neuropsychiatric disorders other than stroke (e.g., anxiety, major depressive disorder, schizophrenia, or bipolar disorder); (3) unstable medical conditions, such as severe atrial fibrillation; (4) addiction to tobacco, alcohol, or other drugs; (5) aphasia or cognitive impairments preventing communication and assessment; and (6) prior exposure to transcranial electromagnetic/ ultrasound stimulation.

The inclusion criteria for the HCs were: (1) comparable age, gender, and level of education with the post-stroke participants; (2) right-handedness. The exclusion criteria were: (1) any significant physical or neuropsychiatric disorders; (2) addiction to tobacco, alcohol, or other drugs; and (3) incomplete information.

### 2.2 Behavioral assessments

Prior to MRI scanning, each stroke patient underwent a comprehensive motor performance evaluation using the Fugl-Meyer Assessment (FMA), administered by a qualified rehabilitation therapist. The FMA is a reliable and valid tool with high sensitivity and specificity for evaluating motor function of the affected side limb in stroke patients (29). The FMA scale is divided into two subscales: the Fugl-Meyer Assessment of the Upper Extremity (FMA-UE) and the Fugl-Meyer Assessment of Lower Extremity (FMA-LE), with the maximum scores were 66 and 34 for the upper and lower limbs, respectively.

### 2.3 Multimodal MRI scanning

All MRI data were acquired on a 3.0 T MRI scanner (Philips Healthcare, Netherlands). High-resolution T1-weighted images were acquired using a three-dimensional fast field-echo sequence with the following parameters: repetition time = 9.9 ms, echo time = 4.6 ms, matrix size = 256 × 256, slice thickness = 1 mm, field of view = 256 mm × 256 mm, 192 sagittal slices, voxel size = 1 mm × 1 mm × 1 mm, and flip angle = 8^°^. T2-weighted images were captured using a MultiVane sequence with the following parameters: repetition time = 4,000 ms, echo time = 91 ms, matrix = 230 × 230, slice thickness = 5 mm, field of view = 230 mm × 230 mm, 30 axial slices, voxel size = 1 mm × 1 mm × 5 mm, and flip angle = 90^°^. Resting-state fMRI was scanned using an echo-planar imaging sequence with the following parameters: repetition time = 2,000 ms, echo time = 30 ms, matrix = 64 × 64, slice thickness = 4 mm, field of view = 192 mm × 192 mm, 38 axial slices, 230 volumes, voxel size = 3 mm × 3 mm × 4 mm, flip angle = 90^°^, and scan time = 8 min 08 s. Diffusion tensor images (DTI) were collected using a spin-echo based planar imaging sequence with the following parameters: repetition time = 8,828 ms, echo time = 70 ms, matrix = 224 mm × 224 mm, field of view = 90 × 90, 60 axial slices, voxel size = 2.5 mm × 2.5 mm × 2.5 mm, 32 non-collinear diffusion-weighted gradient directions (*b* = 1,000 s/ mm^2^) and one non-diffusion-weighted images (*b* = 0 s/mm^2^).

### 2.4 Lesion overlap analysis

**Figure 1:**
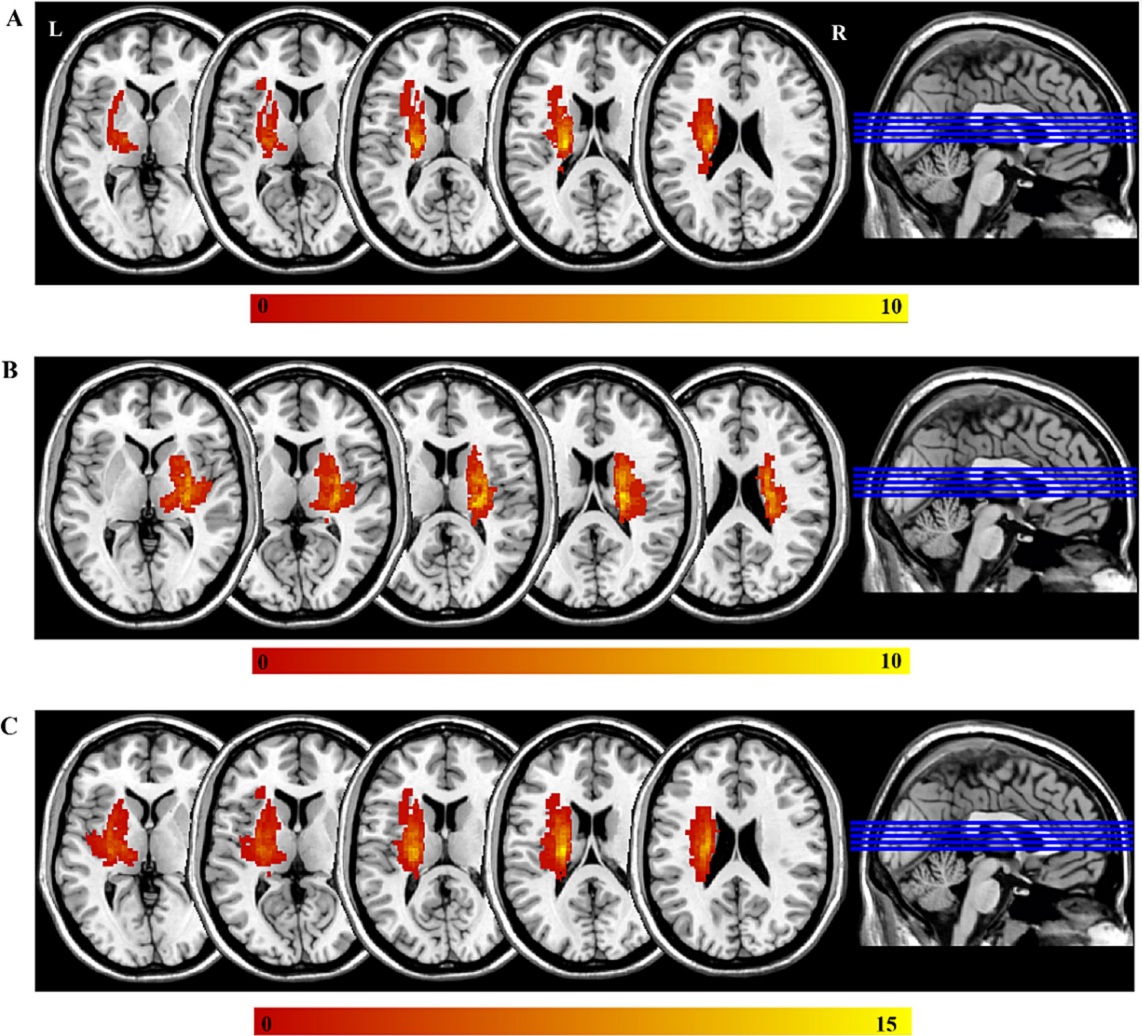
The lesions of all the participants. The color bar represents the number of participants with stroke. **(A)** The left-sided lesions; **(B)** the right-sided lesions; **(C)** the overlapping of all lesions by mirroring the right-side lesions to the left side. L, left; R, right.

First, a physician outlined the lesions on individual T2-weighted images slice by slice using the MRIcron^2^, and a neuroradiologist then confirmed the location and volume of the lesions. Second, individual T2-weighted image was registered to an EPI template (Montreal Neurological Institute space) using affine and nonlinear registration method, which generated a transform matrix that was subsequently applied to the lesion mask for each subject. The precise lesion locations for each patient are displayed in Supplementary Figure S1 and Supplementary Table S3. Finally, all normalized lesion masks were summed to generate a lesion overlap map for each patient subgroup, as illustrated in Figure 1. Throughout the process of identifying the lesions in the MRI data, analysts were blinded to the clinical information.

### 2.5 Image preprocessing

DTI data preprocessing in this study was conducted using FMRIB’s Software Library (FSL) version 4.1^3^, with the following steps: (1) data integrity check; (2) brain segmentation and correction for eddy current-induced distortions; and (3) estimation of diffusional tensor and calculation of fractional anisotropy (FA).

Resting-state fMRI data underwent preprocessed using the Data Processing Assistant for Resting-State fMRI (DPARSF) software version 5.1^4^ (30). Consistent with reports in our previous studies (10, 11), and the steps were as follows: (1) discarded the first 10 image; (2) performed slice-timing correction; (3) corrected for the temporal difference and head motion (exclude data if translation > 2.5 mm or rotation > 2.5^°^ in any direction); (4) calculated the average framewise displacement; (5) aligned individual 3D T1-weighted images with blood oxygenation level depended (BOLD) functional images; (6) segmented gray matter, white matter, and cerebrospinal fluid using T1-weighted images; (7) transformed functional images into MNI standard space and resample to a 3-mm isotropic voxel; (8) smoothed normalized images using an isotropic Gaussian filter at a full width at a half maximum of 6 mm; (9) performed multiple linear regression to remove covariates, including 24 head motion parameters, global mean signal, white matter signal, and cerebrospinal fluid signal (31, 32); (10) applied temporal bandpass filtering (0.01–0.1 Hz).

### 2.6 SC and FC analysis

This study utilized DSI-Studio^5^ to perform deterministic fiber tractography for constructing SC. The process included the following steps: initially, fiber tractography was performed to obtain the corresponding output images of the fiber bundle. Secondly, the whole-brain tractography parameters, as outlined by Baum et al. (33), were set with 1,000,000 seed points, an angle threshold of 45^°^, a step size of 0.94 mm, and the default anisotropy threshold applied. Fiber trajectories were then smoothed by averaging 90% of the current direction with the previous one. Any trajectories that were shorter than 10 mm or exceeding 400 mm were discarded. Finally, b0 diffusion-weighted images were transformed to Montreal Neurological Institute space using affine transformation metrics, and all diffusion-derived metrics underwent spatial transformation into the AAL2 template space^6^. SC between brain regions was quantified using the AAL2 atlas to define region-specific connectivity profiles.

The FC was calculated by using two main steps: first, the BOLD signals from all voxels within each region of interest (ROI) were extracted and averaged to obtain the mean BOLD signal. Second, Pearson correlation analysis was performed on the time series of all the ROIs in the AAL2 atlas, with the resulting correlation coefficient (*r*) defined as the measure of FC.

### 2.7 Statistical analysis

The demographic characteristics and clinical assessments were analyzed using the version 21.0 of Statistical Package for Social Sciences (SPSS). Normality was first analyzed for age, education, and mean head movement. For non-normally distributed data, the Mann–Whitney rank-sum test was applied. For normally distributed data, between-group differences were analyzed using a two-sample *t*-test or a two-sample corrected *t*-test, depending on the homogeneity of variance. Gender was analyzed by the Chi-squared test for differences between groups.

ROI-wise SC and FC differences between post-stroke patients and HCs were analyzed using two-sample *t*-tests in MATLAB 2016a^7^, with age, gender, education level, and mean head motion included as covariates. The significance level was set at *p* < 0.05, and the false discovery rate (FDR) was applied to correct for multiple comparisons.

In addition, Pearson’s correlations between SC and FC in ROI-ROIs showing both SC and FC alterations, as well as the FMA (FMA-UE, FMA-LE) score, were calculated. A significance level of *p* < 0.05 was considered statistically significant. And the false discovery rate (FDR) was utilized to adjust for Pearson’s correlations.

## 3 Result

### 3.1 Demographic and clinical characteristics following data exclusion

Four eligible participants were excluded due to incomplete DTI scans for personal reasons (two HCs) and excessive head motion (two post-stroke participants). Finally, thirty-six non-acute subcortical post-stroke patients and thirty-six HCs, matched for gender, age, education level, and mean head movement, were included in the final analysis. Detailed demographic characteristics and clinical assessments of both post-stroke patients and HCs are displayed in Table 1.

**Table 1:** Demographic characteristics and clinical assessments data of participants in this study.

Baseline characteristics		Stroke participants (*n* = 36)	HCs (*n* = 36)	*p*-value
Age (M ± SD, years)^a^		56.72 ± 9.59	58.75 ± 7.68	0.29
Gender (female: male, *n*)^b^		3:33	3:33	1.00
Education (M ± SD, years)^a^		9.89 ± 2.77	9.56 ± 3.00	0.44
Duration of illness (M ± SD, months)		14.22 ± 10.52	-	-
Mean head movement (M ± SD, mm)^a^		0.1 ± 0.16	0.13 ± 0.10	0.06
Lesion side (left: right, *n*)		17:19	-	
Lesion volume (M ± SD, ml)		3.14 ± 2.98	-	-
FMA (M ± SD, scores)	FMA-UE	47.50 ± 18.55	-	-
	FMA-LE	27.64 ± 6.56	-	-

^a^ represents independent *t*-test. ^b^ represents Chi-Square test. *n*, number; M, mean; SD, standard deviation; FMA, the Fugl-Meyer Assessment; FMA-UE, the Fugl-Meyer Assessment Upper Extremity Scale; FMA-LE, the Fugl-Meyer Assessment Lower Extremity Scale.

### 3.2 Significant differences in SC and FC between post-stroke patients and HCs

Compared with HCs, patient group showed significant decreases in several SCs, including connections between the right precentral gyrus and right middle frontal gyrus, between the right superior frontal gyrus and right supplementary motor area, between the right supplementary motor area and right middle cingulate gyrus, and so on (See Supplementary Table S1 for more details). Notably, the FCs among regions abovementioned did not showed significant differences between groups.

We also observed significant decreases in FCs but no changes in SCs in patient group, such as connections between the left middle cingulate gyrus and left posterior cingulate gyrus, between the right middle frontal gyrus and left parahippocampal gyrus, between the left middle cingulate gyrus and left calcarine fissure and surrounding cortex, and so on (See Supplementary Table S2 for more details).

**Figure 2:**
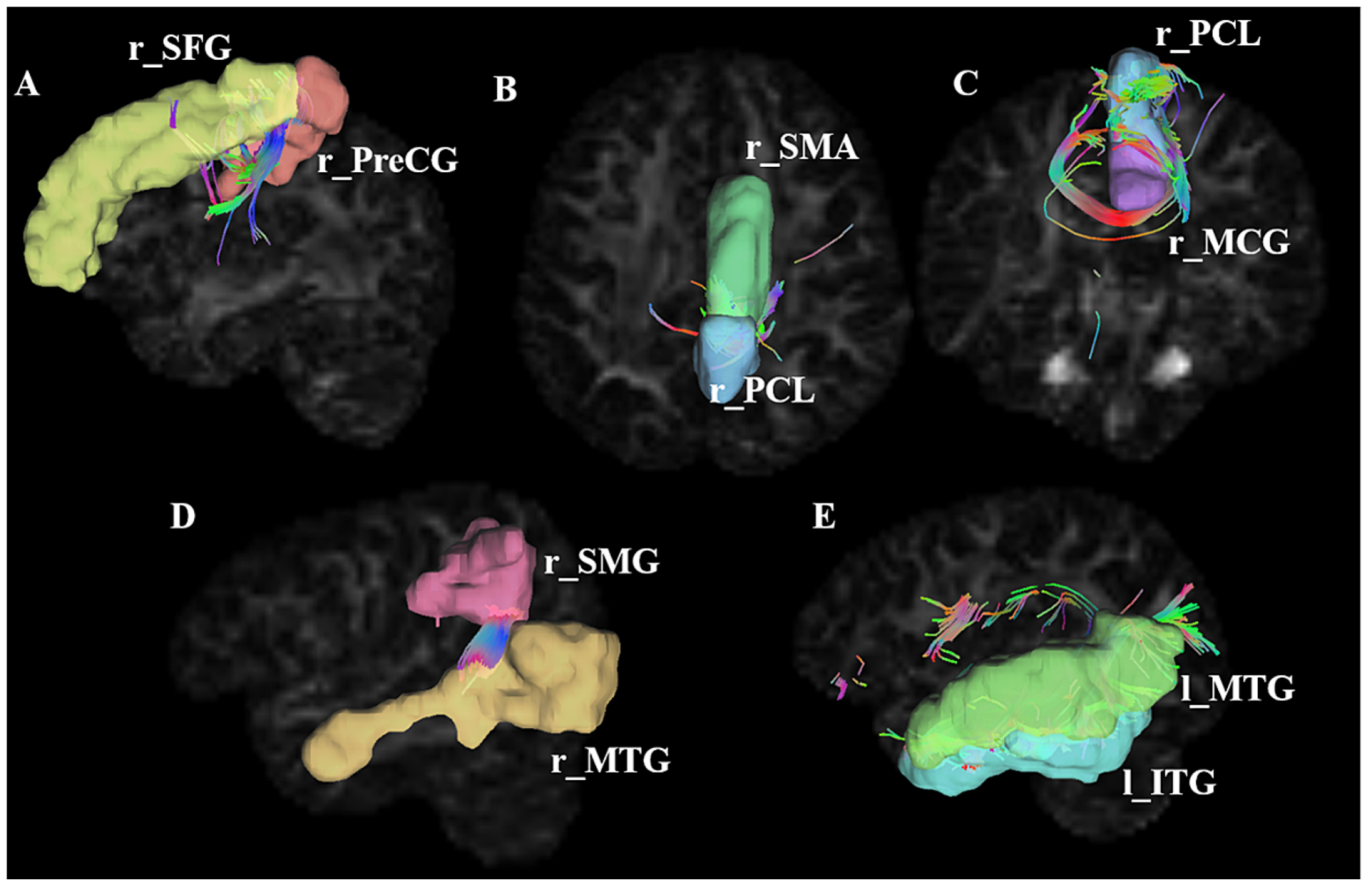
Connections showing significant alterations in SC alterations in stroke patients. **A–E** represents significant SC alterations in stroke patients between r_SFG and r_PreCG, between r_SMA and r_PCL, between r_PCL and r_MCG, between r_SMG and r_MTG, between l_MTG and l_ITG, respectively. ROI, region of interest; PreCG, precentral gyrus; SFG, superior frontal gyrus; SMA, supplementary motor area; PCL, paracentral lobule; SMG, superior marginal gyrus; MTG, middle temporal gyrus; ITG, inferior temporal gyrus; l, left; r, right. The number of connecting lines in the figure represents the strength of the structural connections; the color of the fiber bundle indicates the direction of fiber tract, where red represents fibers running left–right, green represents fibers running anterior–posterior, and blue represents fibers running superior–inferior.

Interestingly, both SC and FC showed significant decreases in post-stroke patients relative to HCs in five paired ROIs (Figure 2; Table 2), including the right PreCG and the right SFG, the right SMA and the right paracentral lobule (PCL), the right middle cingulate gyrus (MCG) and the right PCL, the right superior marginal gyrus (SMG) and the right MTG, and the left MTG and the left inferior temporal gyrus (ITG). However, these differences between groups did not survive FDR correction of *p* < 0.05.

**Table 2:** Inter-group differences in both SC and FC between post-stroke patients and HCs.

ROI-ROI		*t*-SC	*p*-SC	*t*-FC	*p*-FC
PreCG.R	SFG.R	−3.74	<0.001	−1.79	<0.001
SMA.R	PCL.R	−4.74	<0.001	−3.39	<0.001
MCG.R	PCL.R	−3.73	<0.001	−3.36	<0.001
SMG.R	MTG.R	−3.04	<0.001	−2.39	<0.001
MTG.L	ITG.L	−3.19	<0.001	−2.95	<0.001

ROI, region of interest; SC, structural connectivity; FC, functional connectivity; PreCG, precentral gyrus; SFG, superior frontal gyrus; SMA, supplementary motor area; PCL, paracentral lobule; SMG, superior marginal gyrus; MTG, middle temporal gyrus; ITG, inferior temporal gyrus; L, left; R, right.

### 3.3 Correlations between SC/FC value and motor performances in post-stroke patients

**Figure 3:**
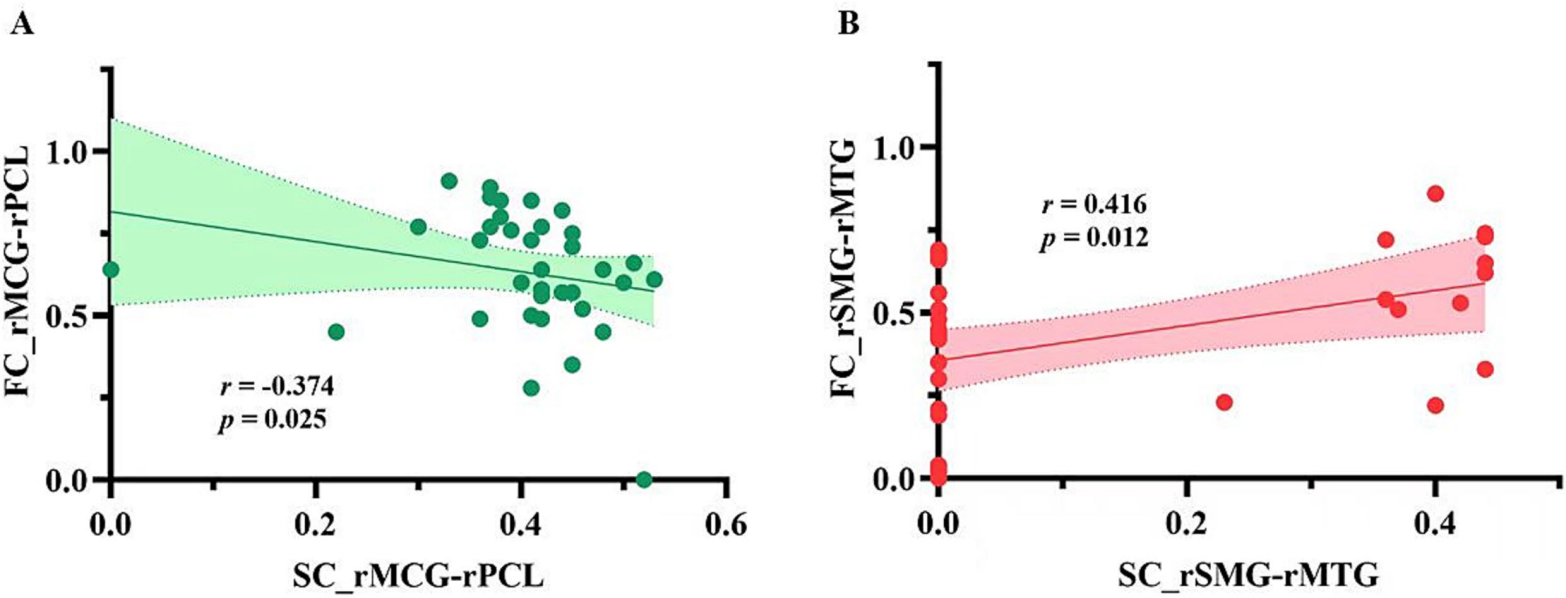
The correlations between SC and FC in ROI-ROI with both SC and FC alterations in patient group. **(A)** Correlation between structural and functional connectivity between the right middle cingulate gyrus and the right paracentral lobule; **(B)** Correlation between structural and functional connectivity between the right supramarginal gyrus and the right middle temporal gyrus. SC_rMCG-rPCL, structural connectivity between the right middle cingulate gyrus and the right paracentral lobule; FC_rMCG-rPCL, functional connectivity between the right middle cingulate gyrus and the right paracentral lobule connections; SC_rSMG-rMTG, structural connections between the right superior marginal gyrus and the right middle temporal gyrus; FC_rSMG-rMTG, functional connections between the right superior marginal gyrus and the right middle temporal gyrus.

**Figure 4:**
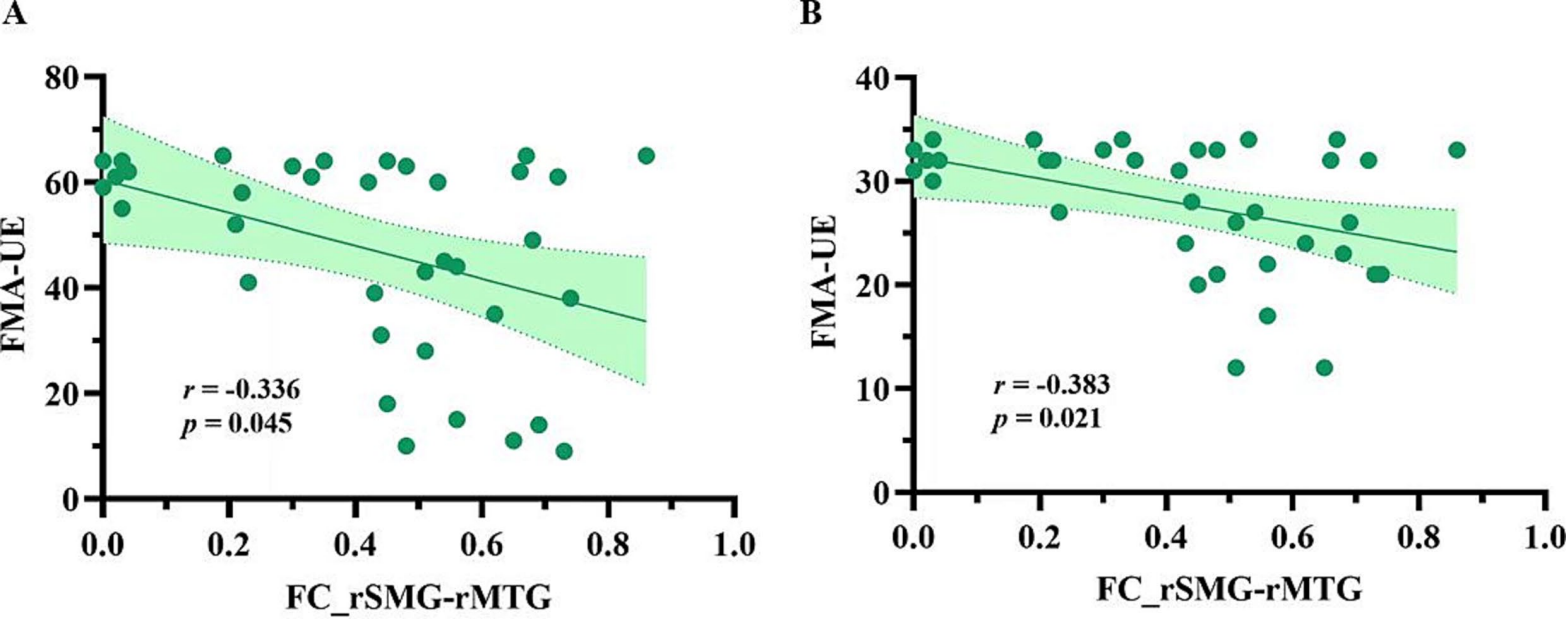
The FCs correlated with motor function in post-stroke patients. **(A)** Correlation between the FC_rSMG-rMTG and the motor function of the upper limb on the hemiplegic side; **(B)** Correlation between the FC_rSMG-rMTG and the motor function of the lower limb on the hemiplegic side. FC_rSMG-rMTG, Functional connectivity between the right superior marginal gyrus and the right middle temporal gyrus; FMA-UE, the Fugl-Meyer Assessment of Upper Extremity; FMA-LE, the Fugl-Meyer Assessment of Lower Extremity; ROI, region of interest.

Figure 3 presents a negative correlation between SC and FC in the right MCG and the right PCL (*r* = −0.374, *p* = 0.025), and a positive correlation between SC and FC in the right SMG and the right MTG (*r* = 0.416, *p* = 0.012). However, these correlations did not survive FDR correction. As shown in Figure 4, among the five pairs of ROI-ROI exhibiting both SC and FC alterations, the FC between the right SMG and the right MTG showed negative correlations with FMA-UE (*r* = −0.336, *p* = 0.045) and FMA-LE (*r* = −0.383, *p* = 0.021) scores across post-stroke patients (did not survive FDR correction). No significant correlation was found between SC and the FMA/FMA-UE/FMA-LE scores in the post-stroke patients.

## 4 Discussion

This study explored the SC and FC alterations, and their correlation with motor performance in non-acute subcortical post-stroke patients. Consistent with our hypothesis, SC and FC alterations were observed not only in sensorimotor-related brain regions but also in high-order brain regions. Additionally, FC between the right SMG and the right MTG was found to be negatively correlated with the motor performance of the affected upper and lower limbs in post-stroke patients.

### 4.1 Neuroplasticity in post-stroke patients with motor dysfunction

Recent researches have highlighted structural and functional neuroplasticity in sensorimotor-related and high-order regions in post-stroke patients. For example, Miao et al. reported a significant increase in the gray matter volume of the ipsilesional SMA in chronic stroke patients with good recovery, along with an increased FC between the ipsilesional MTG and SMG (34). Similarly, Kalinosk et al. identified significant decreases in SC and FC within the prefrontal cortex, posterior parietal cortex, and cingulate gyrus in chronic stroke patients (26). In patients with chronic pontine infarction, Xue et al. showed significantly reduced SC in the contralesional PreCG compared to the ipsilesional hemisphere (35). In a study from Astrakas et al., a notable decline of nodal efficiency was observed in the left SMG in patients with chronic-phase stroke (36). Additionally, our previous study found significantly increased FC between the right SFG, left ITG, left PreCG, and the cerebellum anterior lobe, as well as between the left PreCG, bilateral MCG, and the cerebellum posterior lobe in non-acute subcortical stroke patients (37). Consistent with these findings, the present study revealed that brain regions showing both SC and FC alterations in non-acute subcortical stroke patients with motor disorders extended beyond the PreCG, SMA, and PCL to include the SFG, SMG, MCG, MTG, and ITG, which suggests that neuroplasticity in both motor-related and high-order regions persist in on-acute post-stroke patients with motor dysfunction. More importantly, we found a negative correlation between SC and FC in the right MCG and PCL, and a positive correlation between the right SMG and MTG. This suggests a strong structural and functional coherence between the SMG and the MTG, while SC and FC between the MCG and PCL appear to change in opposite directions, indicating SC and FC change differently across distinct pairs of brain regions in the chronic stage. Notably, SC was absent between the right SMG and the right MTG in some post-stroke patients, which may be due to the limitations of fiber tractography approach used in the present study. Therefore, future studies should consider employing additional analytical techniques to more comprehensively capture SC changes.

### 4.2 Roles of the SMG and MTG in motor function in post-stroke patients

The SMG plays a crucial role in regulating cognitive motor function (38), including hand writing (39), attention (40) and spatial perception (41). As functional recovery progresses in post-patients, changes in the functional activity of the SMG are observed (42). Consistent with the findings of the present study, previous research has identified significant correlation between functional reorganization and motor function in the SMG among post-stroke patients. For instance, Martin et al. found that instrumental-related motor function was positively correlated with functional activity in the SMG in chronic-phase post-stroke patients (38). Our previous study on cortical morphology in patients with motor deficits after non-acute subcortical stroke demonstrated a negative correlation between the volume of the right SMG and the motor function of the affected upper and lower limbs (12). Taken together, these findings suggest that structural and functional alterations in the SMG are involved in motor-related function in post-stroke patients. The MTG is involved in high-order motor control (43). Previous studies have found negative correlations between functional reorganization or structural alterations in the MTG and motor function in post-stroke patients, which aligns with the findings of the present study. For example, Zhao et al. reported that effective connectivity between the ipsilesional MTG and M1 was negatively correlated with total FMA scores (44). Similarly, Wu et al. observed a negative correlation between gray matter density in the left MTG and functional deficits in ischemic stroke patients (45). These findings indicate that the functional reorganization and structural remodeling of the MTG may play a role in motor deficits in post-stroke patients. In the present study, we identified a positive correlation between SC and FC in the right SMG and MTG. These two higher-order motor regulatory areas not only exhibit structural and functional alterations, but also show a trend of negative correlation between FC and motor function of the hemiplegic side in post-stroke patients. This suggests that the SC and FC alterations in these regions may be implicated in the motor function impairment observed in post-stroke patients during the non-acute phase.

### 4.3 Limitations

First, the present study employed a cross-sectional observational design, which limits our ability to dynamically track the SC and FC alterations and their correlation with motor function changes over time in non-acute stroke patients. Longitudinal studies are necessary to investigate how SC and FC alterations evolve as the disease progresses. Second, the sample size was relatively small, and there was a heavy male predominance in the present study. Although we controlled for gender as a nuisance covariate in the statistical analysis, a study with a larger sample size and the appropriate gender ratio would be necessary to confirm our findings in the future. Third, we found a negative correlation between the FC in the right SMG and the right MTG with the motor function of the affected upper and lower limbs in post-stroke patients. However, these correlations did not survive FDR correction, likely due to the limited sample size of the study. Fourth, to control for lesion location, we included only unilateral stroke patients with lesions predominantly in subcortical regions, such as the radial crown and basal ganglia regions. Although lesions were employed as a covariate in SC and FC comparisons between post-stroke patients and HCs, the side of the lesion may still affect the results. Future research should explore structural and functional dysconnectivity in subgroups based on lesion laterality. Fifth, although this study focused on cortical SC analyses in stroke patients with subcortical lesions, and incorporated lesion volume as a covariate in intergroup comparisons, we acknowledge a methodological limitation in not systematically accounting for potential long-range diaschisis effects of subcortical lesions on cortical SC metrics. Finally, this study lacked FLAIR imaging, preventing white matter hyperintensity quantification. Future work will include FLAIR scans to assess the effects of white matter hyperintensity on neural and behavioral measures.

## 5 Conclusion

The present study, utilizing multimodal MRI in non-acute subcortical post-stroke patients with hemiplegia, demonstrated changes in both SC and FC in the sensorimotor-related and the high-order brain regions. Notably, among the regions exhibiting both SC and FC alterations, we found a positive correlation between SC and FC in the MCG and the PCL, while a negative correlation between SC and FC in the SMG and the MTG. In addition, the FC between the right SMG and MTG was negatively correlated with the motor function in the affected limbs. These findings suggest that structural and functional dysconnectivity may be associated with motor function impairment in patients with non-acute subcortical stroke and provide novel insights and directions for targeted rehabilitation interventions.

## Data Availability

The raw data supporting the conclusions of this article will be made available by the authors, without undue reservation.

## Footnotes

^1^ http://www.gpower.hhu.de

^2^ http://www.nitrc.org/projects/mricron

^3^ http://www.fmrib.ox.ac.uk/fsl

^4^ http://rfmri.org/DPARSF

^5^ https://dsi-studio.labsolver.org

^6^ http://www.gin.cnrs.fr/AAL2

^7^ https://ww2.mathworks.cn/products/matlab.html

## Glossary

SC: structural connectivity
FC: functional connectivity
SFG: superior frontal gyrus
SMA: supplementary motor area
M1: primary motor cortex
PreCG: precentral gyrus
MTG: middle temporal gyrus
HCs: healthy controls
FMA: Fugl-Meyer Assessment
FMA-UE: Fugl-Meyer Assessment of the Upper Extremity
FMA-LE: Fugl-Meyer Assessment of Lower Extremity
DTI: Diffusion tensor images
FA: fractional anisotropy
BOLD: blood oxygenation level depended
ROI: region of interest
SPSS: Statistical Package for Social Sciences
FDR: false discovery rate
PCL: paracentral lobule
MCG: middle cingulate gyrus
SMG: superior marginal gyrus
ITG: inferior temporal gyrus
