# Supplementary material for: Structural and functional disconnections in non-acute post-stroke patients
Source: Front Neurol. 2025 Jun 25;16:1542292. doi: 10.3389/fneur.2025.1542292 (PMC12237644; doi:10.3389/fneur.2025.1542292)
Supplement: Supplementary file 1 [file Supplementary_file_1.docx]

**Supplementary Methods and Results**

We applied a linear regression model to predict the motor scores assessed by FMA-UE/FMA-LE and explore the importance of alterations in SC and FC. The model examined the relationship between neuroimaging features and motor assessment outcomes. Specifically, we examined the SC/FC matrices between AAL2 atlas-defined ROIs, with the FMA-UE/FMA-LE scores serving as the dependent variable. The resultant regression models can be used to predict the motor recovery outcome of a patient by using the SC/FC of the patient. By plotting the predicted values of an FMA-UE score (FDR corrected p < 0.001, SC: β = -64.96, FC: β = 10.38), we found that SC and FC between the left and right paracentral lobules showed the performance of the significant predictive models.

**Supplementary Figure**
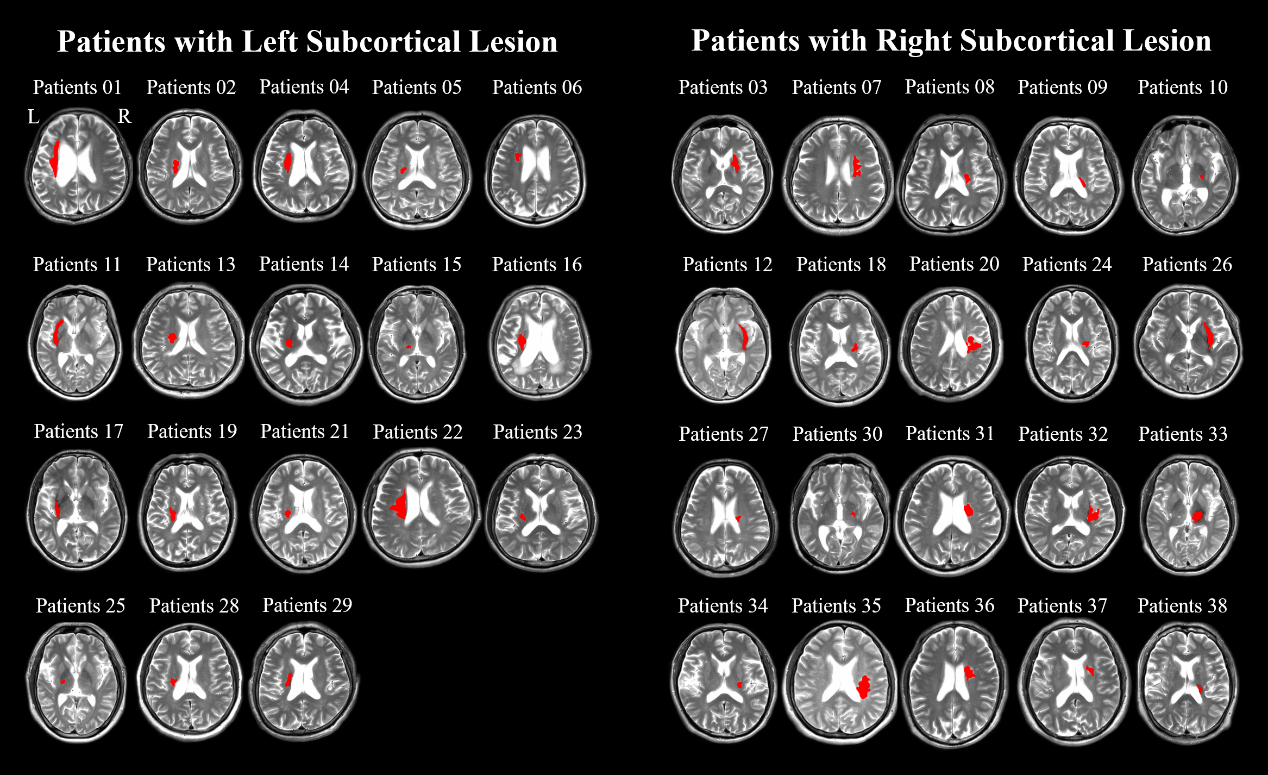


**Supplementary Figure. Lesion display for each patient.** The red region represents individual lesion.

**Supplementary Tables**

**Table S1.** Inter-group differences in only SC between stroke patients and HCs.

| ROI-ROI | | *t*-SC | | *P*-SC |  |
| --- | --- | --- | --- | --- | --- |
| PreCG.R | | MFG.R | | -3.37 | <0.001 |
| SFG.R | | SMA.R | | -3.84 | <0.001 |
| SFG.L | | SFGmed.L | | -2.97 | 0.003 |
| SMA.R | | MCG.R | | -5.15 | <0.001 |
| PCG.L | | PHG.L | | -2.96 | 0.003 |
| CAL.R | | CUN.R | | -3.65 | <0.001 |
| CUN.R | | SOG.R | | -3.75 | <0.001 |
| SOG.L | | MOG.L | | -2.96 | 0.003 |
| PreCG.R | | PoCG.R | | -4.06 | <0.001 |
| PoCG.L | | SPG.L | | -3.39 | <0.001 |
| SPG.L | | IPG.L | | -3.92 | <0.001 |
| IPG.L | | SMG.L | | -3.21 | 0.001 |
| IPG.L | | ANG.L | | -3.53 | <0.001 |
| SPG.L | | PCUN.L | | -3.27 | <0.001 |
| SPG.R | | PCUN.R | | -3.61 | <0.001 |
| PHG.R | | PCL.R | | -3.23 | 0.001 |
| PoCG.R | | PCL.R | | -3.14 | 0.002 |
| SMA.R | | CAU.R | | -3.74 | <0.001 |
| PoCG.R | | PUT.R | | -3.78 | <0.001 |
| PoCG.R | | PAL.R | | -4.76 | <0.001 |
| PCL.R | | PAL.R | | -3.90 | <0.001 |
| PUT.R | | PAL.R | | -3.19 | 0.001 |
| PAL.L | | THA.L | | -3.03 | 0.002 |
| PCL.R | | THA.R | | -3.22 | 0.001 |
| PUT.R | | THA.R | | -3.50 | <0.001 |
| PAL.R | | THA.R | | -4.62 | <0.001 |
| PAL.R | | PAL.R | | -3.60 | <0.001 |
| PAL.R | | PAL.R | | -3.06 | 0.002 |
| PAL.R | | PAL.R | | -3.63 | <0.001 |

Note：ROI, region of interest; SC, structural connectivity; FC, functional connectivity; PreCG, precentral gyrus; MFG, middle frontal gyrus; SFG, superior frontal gyrus; SMA, supplementary motor area; SFGmed, superior frontal gyrus, medial; MCG, middle cingulate gyrus; PCG, posterior cingulate gyrus; PHG, parahippocampal gyrus; CAL, calcarine fissure and surrounding cortex; CUN, cuneus; SOG, superior occipital gyrus; MOG, middle occipital gyrus; PoCG, postcentral gyrus; SPG, superior parietal gyrus; IPG, inferior parietal gyrus; SMG, supramarginal gyrus; ANG, angular gyrus; PCUN, precuneus; PCL, paracentral lobule; CAU, caudate nucleus; PUT, lenticular nucleus, putamen; PAL, lenticular nucleus, pallidum; THA, thalamus; L, left; R, right.

**Table S2.** Inter-group differences in in FC between stroke patients and HCs.

| ROI-ROI | | *t*-FC | *p*-FC |
| --- | --- | --- | --- |
| MCG.L | PCG.L | -4.47 | <0.001 |
| MFG.R | PHG.L | -4.37 | <0.001 |
| MCG.L | CAL.L | -5.04 | <0.001 |
| MCG.L | CUN.L | -4.68 | <0.001 |
| MCG.L | LING.L | -4.85 | <0.001 |
| MCG.L | LING.L | -4.77 | <0.001 |
| MCG.R | LING.R | -4.37 | <0.001 |
| SMA.L | SOG.L | -4.71 | <0.001 |
| MCG.L | SOG.L | -4.80 | <0.001 |
| MCG.L | SOG.L | -4.56 | <0.001 |
| MCG.L | FFG.L | -4.29 | <0.001 |
| MCG.R | FFG.L | -4.46 | <0.001 |
| MCG.L | FFG.L | -4.37 | <0.001 |
| MCG.R | FFG.L | -4.31 | <0.001 |
| MCG.R | FFG.L | -4.29 | <0.001 |
| FFG.L | FFG.L | -4.65 | <0.001 |
| IOG.L | SMG.L | -4.63 | <0.001 |
| FFG.L | SMG.R | -5.04 | <0.001 |
| MCG.R | THA.L | -4.72 | <0.001 |
| MCG.L | THA.R | -4.51 | <0.001 |
| MFG.R | MTG.L | -4.77 | <0.001 |
| IFGtriang.L | MTG.L | -4.39 | <0.001 |
| IFGoperc.R | MTG.R | -4.34 | <0.001 |
| PCUN.R | CP.R | -4.45 | <0.001 |
| SFG.R | CH.L | -4.71 | <0.001 |
| ROL.R | CH.L | -4.42 | <0.001 |
| MCG.L | CH.L | -4.65 | <0.001 |
| ROL.R | CH.L | -4.85 | <0.001 |
| MFG.R | CH.L | -4.60 | <0.001 |
| SFGmed.L | CH.L | -4.93 | <0.001 |
| SFGmed.R | CH.L | -4.29 | <0.001 |
| MCG.L | CH.L | -4.38 | <0.001 |
| MFG.R | CH.R | -4.37 | <0.001 |
| SFGmed.L | CH.R | -4.76 | <0.001 |
| SFGmed.R | CH.R | -4.61 | <0.001 |
| SFG.L | CV | -4.33 | <0.001 |
| ROL.R | CV | -4.95 | <0.001 |
| SFGmed.R | CV | -4.49 | <0.001 |

Note：ROI, region of interest; SC, structural connectivity; FC, functional connectivity; MCG, middle cingulate gyrus; PCG, posterior cingulate gyrus; MFG, middle frontal gyrus; PHG, parahippocampal gyrus; CAL, calcarine fissure and surrounding cortex; CUN, cuneus; LING, lingual gyrus; SMA, supplementary motor area; SOG, superior occipital gyrus; FFG, fusiform gyrus; IOG, inferior occipital gyrus; SMG, supramarginal gyrus; THA, thalamus; MFG, middle frontal gyrus; MTG, middle temporal gyrus; IFGtriang, inferior frontal gyrus, triangular part; IFGoperc, inferior frontal gyrus, opercular part; PCUN, precuneus; SFG, superior frontal gyrus; ROL, rolandic operculum; SFGmed, superior frontal gyrus, medial; CH, cerebellar hemispheres; CV, cerebellar vermis; CP, cerebellar peduncles; L, left; R, right.

**Table S3.** Lesion location of patients in the stroke group

| Number of subjects | Side of the lesion | Location of the lesion |
| --- | --- | --- |
| Subject 01 | Left | Corona radiata |
| Subject 02 | Left | Corona radiata, Basal ganglia |
| Subject 03 | Right | Corona radiata, Basal ganglia |
| Subject 04 | Left | Corona radiata, Basal ganglia |
| Subject 05 | Left | Corona radiata, Basal ganglia |
| Subject 06 | Left | Intimal capsule, Basal ganglia |
| Subject 07 | Right | Corona radiata |
| Subject 08 | Right | Basal ganglia |
| Subject 09 | Right | Basal ganglia |
| Subject 10 | Right | Basal ganglia |
| Subject 11 | Left | Corona radiata |
| Subject 12 | Right | Basal ganglia |
| Subject 13 | Left | Corona radiata, Basal ganglia |
| Subject 14 | Left | Corona radiata, Basal ganglia |
| Subject 15 | Left | Corona radiata, Basal ganglia |
| Subject 16 | Left | Basal ganglia |
| Subject 17 | Left | Corona radiata, Basal ganglia |
| Subject 18 | Right | Basal ganglia |
| Subject 19 | Left | Basal ganglia |
| Subject 20 | Right | Basal ganglia |
| Subject 21 | Left | Corona radiata, Basal ganglia |
| Subject 22 | Left | Corona radiata, Basal ganglia |
| Subject 23 | Left | Corona radiata, Basal ganglia |
| Subject 24 | Right | Corona radiata, Basal ganglia |
| Subject 25 | Left | Corona radiata, Basal ganglia |
| Subject 26 | Right | Corona radiata, Basal ganglia |
| Subject 27 | Right | Basal ganglia |
| Subject 28 | Left | Corona radiata, Basal ganglia |
| Subject 29 | Left | Basal ganglia |
| Subject 30 | Right | Corona radiata |
| Subject 31 | Right | Corona radiata |
| Subject 32 | Right | Corona radiata, Basal ganglia |
| Subject 33 | Right | Basal ganglia |
| Subject 34 | Right | Corona radiata, Basal ganglia |
| Subject 35 | Right | Corona radiata, Basal ganglia |
| Subject 36 | Right | Corona radiata, Basal ganglia |
| Subject 37 | Right | Corona radiata, Basal ganglia |
| Subject 38 | Right | Corona radiata, Basal ganglia |
